# Medical graduates’ preparedness to practice: a comparison of undergraduate medical school training

**DOI:** 10.1186/s12909-017-0859-6

**Published:** 2017-02-06

**Authors:** Susan Miles, Joanne Kellett, Sam J. Leinster

**Affiliations:** 1 Norwich Medical School, University of East Anglia, Norwich, NR4 7TJ UK; 2 Clinical Research and Trials Unit, Norwich Medical School, Norfolk and Norwich University Hospital, University of East Anglia, Norwich, NR4 7TJ UK

**Keywords:** Preparedness, Foundation training, Induction, Undergraduate medical education, F1 doctor, Problem-based learning, PBL

## Abstract

**Background:**

There is evidence that newly qualified doctors do not feel prepared to start work. This study examined views of first year Foundation doctors (F1s) regarding how prepared they felt by their undergraduate medical education for skills required during the first Foundation training year in relation to their type of training.

**Method:**

One-hundred and eighty two F1s completed a questionnaire during their first rotation of Foundation training. Analysis was conducted by type of medical school training: Problem-Based Learning (PBL), Traditional or Reformed.

**Results:**

F1s from medical schools with a PBL curriculum felt better prepared for tasks associated with communication and team working, and paperwork than graduates from the other medical school types; but the majority of F1s from all three groups felt well prepared for most areas of practice. Less than half of graduates in all three groups felt well prepared to deal with a patient with neurological/visual problems; write referral letters; understand drug interactions; manage pain; and cope with uncertainty. F1s also indicated that lack of induction or support on starting work was affecting their ability to work in some areas.

**Conclusions:**

Whilst F1s from medical schools with a PBL curriculum did feel better prepared in multiple areas compared to graduates from the other medical school types, specific areas of unpreparedness related to undergraduate and postgraduate medical training were identified across all F1s. These areas need attention to ensure F1s are optimally prepared for starting work.

**Electronic supplementary material:**

The online version of this article (doi:10.1186/s12909-017-0859-6) contains supplementary material, which is available to authorized users.

## Background

Numerous studies have identified a perception among new medical graduates that they are unprepared for their role as clinicians [1–6]. There are indications that the lack of confidence is not global but is concentrated on some of the skills needed to fulfil the duties of the post [2, 4, 5]. Furthermore, there is evidence of differences between medical schools in how prepared their graduates feel by their undergraduate medical training [7, 8].

Following widespread curriculum reform in recent years UK medical schools can be broadly categorised into 3 groups, which we have designated as traditional, reformed and Problem-based learning (PBL) based on their differing approaches to curriculum design. Medical schools with a predominantly PBL curriculum were designated ‘PBL’; those with a very clear preclinical/clinical divide and chiefly discipline-based teaching in a lecture format were designated ‘Traditional’; and those which have adopted a core integrated curriculum but not adopted PBL as the main teaching method were designated ‘Reformed’ (Additional file 1: Table S1A). Information on the curricula was obtained from school websites and other published material. Norwich Medical School admitted its first cohort of students in 2002, graduating them in 2007. The main learning approach is PBL, in conjunction with the introduction of formal clinical placements in primary and secondary care from year 1. Indeed, clinical placements form the same proportion of teaching in all 5 years of the course. It was imperative that the outcomes of this approach be evaluated, particularly the graduates’ clinical preparedness.

Previous research has shown a beneficial effect of a PBL curriculum on graduate’s preparedness. In a series of articles, researchers at the University of Manchester medical school compared graduates from their old traditional and new PBL-based curricula. They found that, compared to graduates of the traditional course, graduates of the PBL course felt better prepared and more competent in a number of areas, with educational supervisors concurring in some of these areas; had a wider conceptualisation of who constituted their team and the role of the team; and were better at dealing with uncertainty in patient management, recognising when to ask for help and requesting that help [9–11]. Similarly, interviews with graduates from the new PBL curriculum at the University of Liverpool and educational supervisors found that the graduates felt themselves to be well prepared overall, in their practical skills, and in their communication skills. Graduates were confident in dealing with difficult communication situations, and they felt they had acquired useful skills for continuing their learning during postgraduate training. The supervisors felt that the new graduates had improved communication, history taking and clinical skills compared to the previous traditional course graduates. The new graduates were also better at recognising their limitations, asking for help, had improved learning skills and were good team-workers with a better understanding of the roles of different healthcare professionals in the team [12, 13].

Following a large survey with recent graduates across the UK, Cave at al. [14] found that graduates from medical schools with a PBL course were more likely to feel well prepared for the jobs they had undertaken since starting work by their experiences at medical school than graduates from traditional courses. In contrast, comparing graduates from three UK medical schools with different curriculum types, including one with a PBL curriculum, using a variety of data collection methods Illing et al. [15] found that graduates from all three schools identified the same areas of preparedness and areas of weakness/concern. Where there were differences, such as the PBL course graduates feeling more prepared for self-directed learning and being more willing to ask for help, it was unclear whether this was due to the type of medical training or to existing personal characteristics [4].

In order to investigate the clinical preparedness of Norwich Medical School’s graduates a series of research studies were undertaken, including a questionnaire study asking about perceived preparedness aimed at all first year Foundation doctors (F1s) in the local East Anglian Foundation School (EAFS). The original intention was to compare the performance of Norwich Medical School graduates with that of graduates from each of the other types of curricula. However, when the data were analysed, the other PBL schools had results similar to Norwich Medical School’s so these data were combined for the final comparisons. Thus, this study examined the views of F1s with regard to how prepared they felt by their undergraduate medical education for various skills required during the first Foundation training year, and compared the perceived preparedness of graduates from three training types. The aim was to identify the areas of concern for F1s and investigate if there were any differences according to type of undergraduate training.

## Methods

### Sample

The study population comprised of all 312 doctors currently employed on F1 posts through the EAFS at 12 NHS Trusts (13 hospitals) in the East of England region. This was a convenience sample based on practical logistics. No effort was made to carry out purposive sampling for any of the graduates’ characteristics. Because this was an exploratory study no power calculations were made but efforts were made to maximise the response rate by offering several options for completion of the questionnaire (see Data collection below). Additionally, as an incentive, participating F1s were given the opportunity to be entered into a prize draw to win one of five £50 vouchers from a well-known internet retailer if they wished.

### Questionnaire design

A questionnaire was designed to investigate how well F1s felt their undergraduate training had prepared them for the first year of Foundation training. The starting point for the questionnaire was the ‘“Fit for Purpose” Undergraduate Medical Education Questionnaire’ used by Wall and colleagues to ascertain pre-registration house officers’ perceptions of preparedness [16]. Additional items were added to cover the competencies set out in the Foundation Programme curriculum current at the time of the study. The questionnaire was piloted with 31 F1s at two local hospitals and then refined further for improved clarity where required. The final questionnaire asked F1s to rate “How well or badly do you think your medical student education and training has equipped you to be competent in the following areas?” for 53 items, using a 6 point preparedness scale ranging from “very badly” to “very well”.

Demographic data were collected: gender, age, year of qualification, name of medical school, whether the medical course had been a graduate entry course, and whether the course had included any time shadowing a Foundation doctor. Further items asked participants to rate their confidence that they had the necessary skills and knowledge to do their F1 job on graduating, their general view of how prepared they were by their undergraduate medical education experiences, and how happy they were to be pursuing a career in medicine. Four open-ended items allowed participants to (i) comment on how prepared they had felt in any of the 53 areas, (ii) to list any skills or knowledge they had needed during their F1 post which had not been adequately covered by their medical training, (iii) to generally note any difficulties they had encountered in their daily work due to being unprepared, and (iv) to provide details of how their medical training could have been done differently to prepare them better (questionnaire available in Additional file 2).

### Validity and reliability of the questionnaire

The questionnaire was scrutinised by a lay panel and by undergraduate and postgraduate medical educators including representatives from the EAFS. The consensus view was that the questions provided a satisfactory means of assessing the graduates’ views of their preparedness so confirming that the questionnaire had *face validity. Content validity* was ensured by deriving the questions from the Foundation Programme curriculum and by cross-reference with the areas explored by other authors in the published literature. *Construct validity* was not regarded as an issue as the questions concerned concrete self-perceptions rather than abstract concepts.

The questionnaire was tested for internal consistency using Cronbach’s alpha for the each of the 8 subscales (see Results below).

### Data collection

Data collection was conducted during the first rotation of the 2011/12 F1 year. Based on experiences in the pilot study, where email recruitment for an online questionnaire had resulted in a low response rate, two recruitment methods were used. One of the authors visited teaching sessions at all of the Trusts to introduce the study and hand out hard copies of the questionnaire. F1s were provided with the opportunity to complete the questionnaire at that time or complete it later and post it back to the research team using a provided pre-paid envelope. Online completion of the questionnaire was also provided for F1s who had not been in attendance at the teaching session. The EAFS sent the link to the online questionnaire with the participant information sheet attached to all F1s at the start of the data collection phase, followed by two reminders.

### Exclusion criteria

Participants who had graduated from medical school before 2010 (to minimise the effect of recall bias), those from an overseas medical school, those who had not specified their medical school, or had changed medical school during their training and also any participants who did not complete the questionnaire to the end were excluded from the analysis.

### Data analysis

Non-parametric analysis was used as the data were not normally distributed. The data from the three medical school-type groups (PBL, Reformed, Traditional) were compared using Kruskal Wallis tests, followed by pairwise comparisons using Mann Whitney tests where post-hoc analysis was needed. Mann Whitney tests were also used for comparisons by gender and age group. Data reduction and subscale creation for the 53 preparedness items was performed using Principal Components Analysis with varimax rotation, followed by Cronbach’s alpha reliability analysis. All analysis was conducted using IBM Statistics SPSS 22 for Windows. The open ended comments were subjected to basic content analysis by two of the authors and an assistant to categorise and summarise the data.

### Creating preparedness subscales

Principal Components Analysis (PCA) was performed for the purposes of data reduction to create subscales for subsequent analysis. A second set of data, not reported, was collected during the third rotation of the first Foundation year. There were no significant differences between the two datasets for any of the 53 preparedness items. As a result the two datasets were combined for data reduction analysis, to ensure that a suitable number of responses (*n* = 334) were available for the number of items (*n* = 53). PCA with varimax rotation was performed, with the number of components used to create subscales determined by examination of the eigenvalues above 1, scree plot and face validity of the resulting components; followed by Cronbach’s alpha reliability analysis with examination of alpha following item deletion. This resulted in the creation of 8 subscales, with alphas ranging from 0.79 to 0.92. Reliability was further checked by looking at Cronbach’s alpha for the 8 subscales for each of Rotations 1 (0.78 to 0.93) and 3 (0.77 to 0.91) separately to ensure that the subscales were indeed appropriate for both sets of data, which they were. Subsequent analysis on the preparedness items was conducted using these 8 subscales for the Rotation 1 data. The items making up each subscale can be seen in Additional file 1: Table S1B of the document.

## Results

### Sample characteristics

Two-hundred thirteen (68% response rate) F1s completed the questionnaire during their first rotation (153 completed in the teaching session, 13 returned by post, 47 completed online). Questionnaires were received from F1s working at all of the 12 Trusts. After applying the pre-determined exclusion criteria there were 182 participants (58% response rate) available for analysis.

The demographic background information can be found in Table 1. At the time of the study, almost all of the participants in all three groups had recently graduated in 2011.

**Table 1:** Background information

Participant background information		PBL *n* = 78	Reformed *n* = 41	Traditional *n* = 63	All *n* = 182
Gender	Male	23 (29.5%)	17 (41.5%)	37 (59%)	77 (42%)
	Female	55 (70.5%)	24 (58.5%)	26 (41%)	105 (58%)
Age	Mean (SD)	26.81 (4.69)	25.98 (3.13)	25.95 (3.26)	26.32 (3.91)
	Range	23–42	23–35	24–38	23–42
Graduate entry course	Yes	4 (5%)	16 (39%)	10 (16%)	30 (16.5%)
Shadowing during medical training	Yes	73 (94%)	37 (90%)	61 (97%)	171 (94%)
Year of graduation	2011	72 (92%)	40 (98%)	60 (95%)	172 (95%)

### Confidence and general preparedness on starting F1 posts

The F1s felt well-prepared by their medical school experience, with no differences between graduates from the three medical school types or between men and women; younger graduates (≤24 years) did feel more prepared than the older graduates. In contrast, the F1s did not feel confident that they possessed the necessary skills and knowledge when they started their F1 year. Men were more confident than women and younger graduates more confident than older graduates in both their knowledge and skills. Medical school type had no effect on confidence in skills but graduates from traditional schools were more confident about their knowledge, although their confidence was still only moderate. Women and younger graduates were happier to have chosen medicine as a career (Additional file 1: Table S1C).

### Subscales results

Eight subscales were identified from the 53 individual items (Table 2 and Additional file 1: Table S1B). The degree to which F1s felt prepared varied between subscales. All F1s felt best prepared for the *Communication and team working* and *Dialoguing with patients* and all least well prepared for the *Paperwork* subscale. However, there were significant differences between the three groups for *Communication and team working*, and *Paperwork*. In both cases graduates from PBL medical schools felt more prepared than graduates from the other two school types. There were no differences for the other 6 preparedness subscales, indicating that F1s who had graduated from the various medical schools felt equally prepared. There were no differences by age for any of eight subscales, and the only gender difference was for the *Dialoguing with patients* subscale where females (mean (SD) = 5.29 (0.60)) felt significantly better prepared than males (mean (SD) = 5.09 (0.66); Mann Whitney, *p* < 0.05).

**Table 2:** Mean (standard deviation) ratings of preparedness (1 = Very badly prepared, 6 = Very well prepared)

Preparedness Subscale (Cronbach’s alpha)	PBL	Reformed	Traditional
Treatment (0.93)	4.69 (0.65)	4.48 (0.56)	4.31 (0.86)
Independent, responsible working (0.92)	4.65 (0.83)	4.54 (0.73)	4.62 (0.90)
Dialoguing with patients (0.91)	5.33 (0.55)	5.11 (0.58)	5.16 (0.75)
History, examination, diagnosis & investigation (0.88)	4.78 (0.62)	4.65 (0.55)	4.82 (0.71)
Communication and team working (0.92) *	5.53 (0.58)	5.30 (0.53)	5.24 (0.80)
Procedural skills (0.88)	5.03 (0.77)	5.00 (0.70)	5.00 (0.91)
Patient safety, ethics and legal issues (0.84)	5.17 (0.65)	4.99 (0.56)	5.16 (0.81)
Paperwork (0.78) *	4.55 (0.79)	3.97 (0.88)	4.15 (0.89)

* Kruskal-Wallis, *p* < 0.05

Although the mean scores for the subscales showed an acceptable level of preparedness in all of the domains, there were differences between individual items within the domains. An arbitrary value of 70% of graduates reporting that they were well or very well prepared was taken as indicating that training for that item had been satisfactory in preparing the F1s. Fewer than half of graduates feeling well-prepared was regarded as an indication of inadequate training. On these criteria, the training for 22/53 items was satisfactory at all types of medical school. For a further 11 items there were differences in the reported levels of preparedness between school types with 1 or 2 groups reporting that they felt well or very well prepared in contrast to the other groups (Table 3), suggesting variability in training between school types.

**Table 3:** Items in which some school types had more students reporting that they were well-prepared

Subscale	Item	School type
Treatment	Dealing with a patient with breathing problems e.g. acute asthma, pulmonary embolism	PBL, Traditional
	Suggesting appropriate treatment for common symptoms e.g. nausea, pain etc.	Reformed
	Dealing with a patient with airway problems	Reformed
	Responding effectively to emergencies	PBL
Independent, responsible working	Teaching colleagues/students	Traditional
	Being responsible for self-directed lifelong learning and professional development	PBL, Traditional
Dialoguing with patients	Discussing medication, including unwanted effects, with patients	PBL, Traditional
History, examination, diagnosis and investigation	Interpreting investigations	PBL
Procedural skills	Prescribing drugs and treatments (including oxygen and fluids) appropriately and clearly	PBL
Patient safety, ethics and legal issues	Understanding the legal framework of medical practice	PBL, Traditional
Paperwork	Keeping an accurate and pertinent medical record	PBL

The School Type shown had ≥70% graduates reporting that they felt well-prepared. The other Schools had <70%

There were 5 items where ≤ 50% of graduates from all 3 groups felt well or very well prepared suggesting that training in these areas was unsatisfactory in all types of school (Table 4). For a further 8 items, ≤50% graduates from 1 to 2 groups felt prepared (Table 5). Notably, the graduates of PBL schools were more likely to feel prepared on the majority of these items than graduates from the other types of school.

**Table 4:** Elements of practice where ≤50% of graduates felt well prepared, amongst all graduate type

Subscale	Item
Treatment	Dealing with a patient with neurological/visual problems e.g. seizures, coma
	Prompt and effective management of acute and chronic pain
Independent, responsible working	Coping with uncertainty
History, examination, diagnosis and investigation	Understanding drug interactions
Paperwork	Writing referral letters

**Table 5:** Items in which some school types had more students reporting that they were less well prepared

Subscale	Item	School type
Treatment	Dealing with a patient with cognitive impairment e.g. dementia, delirium	Reformed, Traditional
	Dealing with a patient with psychiatric/psychological problems e.g. substance abuse, psychosis	Reformed, Traditional
	Dealing with acutely unwell patients with complex needs e.g. medicine for the elderly	PBL, Traditional
Independent, responsible working	Prioritisation of tasks/time management	Reformed
	Managing your own health, including stress	Reformed, Traditional
History, examination, diagnosis and investigation	Critical use of evidence (e.g. from audit, guidelines and research literature) in diagnosis and/or treatment	Reformed
Paperwork	Completing discharge summaries	Reformed, Traditional
	Verification of death/death certification	Reformed

The School Type shown had ≤50% graduates reporting that they were well-prepared. The other Schools had >50%

The free text comments supported the responses to the closed questions and added further explanation for some items. Task prioritisation and time management were highlighted as particularly problematic areas, with challenges arising from multiple demands from a variety of people at the same time. These issues were especially severe when F1s felt they were working alone at night or at the weekends. In part, this arose from a realisation that they were now responsible for the care of patients and were accountable for the decisions that they made.

Graduates highlighted difficulties which they had encountered in not knowing when to involve their seniors. The item *Seeking help and advice from senior colleagues* had been well rated in terms of preparedness, suggesting that the graduates are prepared to ask for help, but they may be struggling to know when to ask. In line with their answers to the closed questions graduates commented that they felt unprepared for some aspects of patient management, particularly managing acutely unwell patients, dealing with medical emergencies and palliative care. They suggested that these were areas that needed more attention in the undergraduate course as they led to challenges in their day-to-day working as F1s.

Whilst the majority of graduates had felt well or very well prepared for the items regarding *Communicating with colleagues* and *Working effectively in a multidisciplinary team*, they felt that they could be better prepared for dealing with other staff, particularly related to conflict / personality clash situations, staff in other departments and hospital politics. Whilst the item *Handover to colleagues* was not particularly poorly rated in terms of preparedness, some graduates commented that this was an area that had not been adequately covered, including aspects of communicating handover information to other staff and completing handover paperwork.

In addition to these areas which might be mitigated by additional or revised undergraduate training, the graduates also commented on difficulties they had encountered in their daily work which related to inadequate induction by their employing Trust. These were areas which the graduates felt the medical school could not prepare them for, because they were related to practices and procedures local to their employing Trust, or activities which the graduates felt they could only learn through experience on the job. Difficulties arose due to a lack of knowledge about local protocols, including IT and paperwork. Which were exacerbated by a general lack of knowledge about what was expected of them as an F1, in terms of their daily tasks in their current F1 post.

The F1s identified a number of areas that they felt could be improved in their undergraduate training. Overall, they wanted increased clinical experience as an active participant through both their clinical placements and shadowing; they wanted to have more responsibility and more experience of the actual role/daily duties of an F1 doctor. Specifically, they wanted more time on the wards, to be able to get involved more during their placements, to have a defined role, to be attached to a team, and to get on-call experience. They wanted more shadowing of F1s, across multiple specialities; including more shadowing at the hospital they were going to be working in. Specific areas mentioned as warranting more time and attention during undergraduate training included the need for more surgical teaching, more prescribing/pharmacology teaching and practice, more time dedicated to teaching on patient management and exposure to acute medicine, and more practice undertaking clinical procedures. They also wanted more opportunities to perform such practical procedures on patients, rather than mannequins.

## Discussion

Our findings confirm previous reports that newly qualified doctors feel unprepared in several areas of practice, even when they believe that their undergraduate training has prepared them well overall. The areas where they are most confident relate to aspects of communication (*Dialoguing with Patients* and *Communication and Team Working*) and procedural skills. This may be a reflection of the emphasis that has been placed on these domains in recent curricula in response to the General Medical Council’s (GMC) requirements in *Tomorrow’s Doctors 2009* [17]. Graduates from PBL schools scored significantly more highly in *Communication and Team Working*. This is in keeping with findings in the literature [18] and probably results from the prolonged experience of small group learning that characterises PBL curricula.

The graduates felt least well-prepared in the area of *Paperwork* which related to the clerical tasks expected of an F1. Although it was only in *Keeping an accurate and pertinent medical record* that more than 70% of the PBL group felt well-prepared, their perception of their competence was higher than the other school types for *Completing discharge summaries* and *Verification of death/death certificate completion*. As the majority of the PBL group were graduates of Norwich Medical School they had had interactive workshops in their final year on these topics. Clearly, however, more needs to be done in this area across all school types.

Although there was no overall difference between the groups in the other domains, there were differences at the level of individual items. In general, where those differences occur, the PBL group felt better prepared than the other groups. There does not appear to be any systematic explanation for these differences. For example, it is difficult to explain why the PBL group should feel better prepared for *Dealing with a patient with breathing problems* but not for *Dealing with an acutely unwell patient with complex needs*.

This difference in preparedness for specific areas between students from the different types of curricula mainly favouring the medical schools with PBL curriculum is in keeping with the findings of previous research [9–14]. The majority of students in the PBL group came from Norwich Medical School and had been exposed to extensive patient contact from first year. Their clinical confidence may be related to the prolonged patient contact rather than problem-based learning but it is not possible to separate out the effects. This is an issue with other previous research, where curriculum revisions from traditional to PBL undergraduate medical training has also commonly included other simultaneous changes such as increasing the amount of patient contact and introducing shadowing. Cave et al. [14] did find that whilst graduates from PBL courses felt more prepared than graduates from medical schools with traditional courses, the relevance of medical school teaching to working as a doctor and feeling able to get help at work were greater predictors of feeling prepared.

It is notable that the students from the PBL schools have a significantly lower self-rating of knowledge, but a similar perception of their overall preparedness to the other groups. A review of the effects of PBL on competency also found that graduates of PBL course rated themselves as having less medical knowledge than graduates in comparable control groups [19]. Additionally, graduates from a curriculum using PBL as its main learning activity for the first four of its 5 years were concerned about their level of scientific knowledge, a view shared by their educational supervisors, but the graduates could provide no instances of this affecting their ability to perform their role [12, 13]. There is no empirical evidence that the PBL graduates have a lower level of knowledge or that their perceptions about amount of knowledge impacts on their ability to perform their role. It may be that, because the PBL process does not set clear boundaries as to what the students should learn it creates uncertainty in the students’ minds about what comprises necessary knowledge.

The items where graduates from all medical schools feel best prepared are either routine tasks (e.g. history taking, examination skills) or those where there is unlikely to be an immediate measurable effect on the patient outcomes if the doctor performs badly (e.g. discussing treatment options, including relative risks and benefits; agreeing on a satisfactory management plan with the involvement of your patient).

There are specific items in the *Treatment* domain that cause concern to all groups of graduates. Careful consideration should be given to ensure that these topics receive adequate coverage in the undergraduate curriculum. For some, such as neurological problems it may be difficult to ensure enough clinical exposure for all students to enable them to feel confident in dealing with such patients. Other conditions, however, are common (e.g. dementia and pain management) and failure to provide adequate learning opportunities cannot be excused.

For many graduates the issues are related to coping with responsibility and coping with uncertainty which are an intrinsic part of clinical practice. In line with the findings of this study, a parallel qualitative study with Foundation doctors at the same 12 Trusts conducted the previous year [20] found that much of their anxiety related to the sudden transition from observer status as students to carrying responsibility for their decisions and actions as doctors. Closely related to this was uncertainty about when to seek help from seniors and a perception that senior help was not always available when needed. It is not clear that changes to the undergraduate curriculum can directly alter these concerns, and it may be that this is better addressed in the workplace by seniors making themselves more open to being contacted or available, and being more explicit about when they expect to be contacted for assistance.

Some of the difficulties experienced by participants in this study related to unfamiliarity with local procedures within the Trust where they were working. These issues relate to induction within the Trust rather than the undergraduate programme. Although induction at the hospital level appears to be satisfactory there remain deficiencies in departmental induction programmes [21, 22]. Recent research has found that increased time spent on induction is associated with lower anxiety amongst F1s [23]. Additionally, an induction course specifically designed to address areas of apprehension and under-preparedness previously identified by medical students and F1s increased perceived preparedness on starting work as an F1 doctor and it also reduced instances of self-reported critical incidents during the first 4 months of Foundation training [24].

Whilst there may be some local policies with which graduates cannot get direct experience until they take up their F1 post, medical schools, Foundation schools and NHS Trusts employing graduates on F1 posts need to work closely together to maximise the relevancy of learning experiences medical students are provided with regarding the administrative tasks which will be expected of them on graduating, whether they stay at a local Trust or go further afield. Medical schools require information about, for example, handover procedures and associated paperwork; procedures for referrals, discharging patients and care packages; IT systems including patient record systems, procedures for ordering investigations and electronic prescribing tools from NHS Trusts all over the UK so that during their training in these areas medical schools are able to make students aware of the different types of documentation and procedures they might encounter at their particular employing Trust.

The desire expressed by the participants in this study to have had more shadowing, more time being actively involved on the wards, more responsibility, more practice undertaking practical procedures on real patients and so on, is in line with research by Burford et al. [25] which found that many graduates across several UK medical schools reported few instances of hands-on experience during their final year of training, with prescribing experience and opportunities for managing acutely unwell patients being particularly lacking. Burford et al. found that more hands-on experience was associated with increased perceived preparedness, and that whilst experiences with both real and simulated acute care situations in final year influenced preparedness the real life experiences had a greater influence on perceived preparedness [25].

The major deficiency in training identified by the graduates is direct clinical experience. Tomorrow’s Doctors 2009 [17] attempted to address this by requiring that all students undertake a period of student assistantship. Most of the doctors in this study had experienced the early forms of student assistantship but were still asking for more practical exposure, particularly with acutely unwell and complex patients. It is essential that student assistantships actively encourage opportunities for participation, rather than observation, with real patients and ensure that they are providing final year students with practical experiences that reflect the skills they will require in the workplace. A recent study by Braniff et al. [26] found that perceived preparation for starting work and for specific knowledge, skills and tasks required of an F1 were significantly improved following a final year student assistantship. Since this research was conducted at Norwich Medical School, additional teaching and clinical placements have been added to Year 4 in areas related to older people’s medicine and oncology and palliative care, students all undertake ALERT (Acute Life-threatening Events: Recognition and Treatment) and ALS (Advanced Life Support) training in Year 5, the Year 5 student assistantship has doubled in length and a second local elective period has been added to the final year. Further research is required to evaluate the impact on the current student assistantships on perceived preparedness and also actual preparedness of UK graduates.

There are some limitations to the reported study. The study is based on the graduates’ self-perceptions rather than an objective assessment of their competence, nonetheless their perception is concordant with that of their educational supervisors [20]. It is based in one Foundation School, but the respondents are drawn from a wide range of UK medical schools so the results should be generalisable to UK based graduates; however they cannot be applied without modification to graduates from other educational systems.

The GMC’s analysis of the progress of doctors in training from the 2015 National Training Survey indicated that many graduates still do not feel well prepared to start their Foundation post generally and in key specific areas (skills in clinical practical procedures, early management of acutely ill patients and prescribing skills), with a high degree of variability between medical schools [27]. This suggests that the data reported here continue to be relevant to those involved in providing undergraduate and Foundation training as well as those involved in directing and monitoring improvements to that training (including the GMC, medical schools, NHS Trusts, and postgraduate deaneries and Local Education Training Boards through Foundation schools coordinated by the UK Foundation Programme Office). There remains a need to improve training to increase graduate’s perceived preparedness in areas such as drug interactions, pain management and other prescribing related skills; management of acutely unwell patients with complex needs, patients with cognitive and neurological impairments, and palliative care; and to continue to maximise opportunities for medical students to get direct, relevant, hands-on clinical experience with actual patients as a matter of priority to ensure that patient safety is not compromised when graduates begin their Foundation posts.

## Conclusion

Graduates from medical schools with a PBL curriculum felt prepared for more areas than graduates from the other types of medical school; however there were areas where preparedness could be improved amongst graduates from all training-types and the suggestions for how their medical school training could have been improved were similar in all three groups. Thus, despite ongoing attempts to improve how well F1 doctors are prepared for practice, there are still areas of practice for which they feel unprepared across multiple undergraduate training types. The major problem seems to be the step change in responsibility that occurs on graduation and it is incumbent upon medical educators to find ways whereby this can be mitigated. Legal constraints limit the extent to which students can be given clinical responsibility for the care of patients but within these constraints students must be given the opportunity to make meaningful contributions to the management of patients.

## Additional files

Additional file 1: Table S1A. Categories of medical schools; **Table S1B.** Number of Foundation doctors prepared for each skill; and **Table S1C.** Mean ratings of general preparedness, confidence in skills and knowledge and happiness at career choice. (PDF 76 kb)
Additional file 2: Preparation for working as an F1. This file is the full questionnaire used to collect data about preparedness for working as an F1. Data from the questions in Part 1 of the questionnaire are reported in this paper (please cite this paper if you use the questionnaire) and data from the questions in Part 2 of the questionnaire are reported in “Foundation doctors’ induction experiences by Susan Miles, Joanne Kellett, Sam J. Leinster. BMC Medical Education2015. 15:118. DOI: 10.1186/s12909-015-0395-1”. (PDF 690 kb)

## Abbreviations

ALERT: Acute Life-threatening Events: Recognition and Treatment
ALS: Advanced Life Support
EAFS: East Anglian Foundation School
F1 / F1s: First year Foundation doctors
GMC: General Medical Council
PBL: Problem-based learning
PCA: Principal Components Analysis
SD: Standard deviation
