# Supplementary material for: Medical graduates’ preparedness to practice: a comparison of undergraduate medical school training
Source: BMC Med Educ. 2017 Feb 6;17:33. doi: 10.1186/s12909-017-0859-6 (PMC5295184; doi:10.1186/s12909-017-0859-6)
Supplement: Additional file 1: Table S1A. — Categories of medical schools; Table S1B. Number of Foundation doctors prepared for each skill; and Table S1C. Mean ratings of general preparedness, confidence in skills and knowledge and happiness at career choice. (PDF 76 kb) [file 12909_2017_859_MOESM1_ESM.pdf]

Supplementary Tables

Medical graduates' preparedness to practice: A comparison of undergraduate medical school training

Susan Miles, Joanne Kellett, Sam J Leinster

Table A: Categories of medical schools

**Type of School**	**Characteristics**	**Exemplar Schools**
Traditional	Distinct preclinical and clinical phases<br>Mainly discipline-based teaching<br>Mainly lecture-based teaching<br>Limited early patient contact	University of Cambridge<br>University College London<br>Imperial College London
Reformed	System-based teaching<br>Horizontal and vertical integration<br>Lecture-based teaching with some small group work<br>Early patient contact	University of Southampton<br>University of Sheffield<br>University of Leeds
Problem-based learning	Case based small group learning<br>Some lectures<br>Horizontal and vertical integration<br>Early patient contact	University of Manchester<br>University of Glasgow<br>Norwich Medical School

Table B: The number (and percentage of responders for that item) of Foundation doctors who indicated that they felt well or very well prepared for each of 53 skills when asked during Rotation 1.
≥70% well or very well prepared shown in bold. ≤50% well or very well prepared in italics.
Also showing the subscale each item was placed into following the Principal Components Analysis
(6 point scale = Very badly prepared, badly prepared, slightly badly prepared, slightly well prepared, well prepared, very well prepared)

**Preparedness item**	**Subscale**	**PBL (n = 78)**	**Reformed (n = 41)**	**Traditional (n = 63)**
Dealing with a patient with airway problems	1	54 (69%)	**29 (71%)**	42 (69%)
Dealing with a patient with breathing problems e.g. acute asthma, pulmonary embolism	1	**68 (87%)**	27 (66%)	**45 (73%)**
Dealing with a patient with circulation problems e.g. hypotension, sepsis	1	**60 (77%)**	**31 (76%)**	**46 (75%)**
Dealing with a patient with cognitive impairment e.g. dementia, delirium	1	40 (51%)	*13 (32%)*	*21 (34%)*
Suggesting appropriate treatment for common symptoms e.g. nausea, pain etc.	1	53 (68%)	**29 (71%)**	32 (52%)
Dealing with an acutely unwell patient with complex needs e.g. medicine for the elderly	1	*32 (41%)*	21 (51%)	*20 (33%)*
Responding effectively to emergencies	1	**61 (78%)**	(26 (63%)	37 (60%)
Dealing with a patient with neurological / visual problems e.g. seizures, coma	1	*31 (40%)*	*15 (38%)*	*20 (32%)*
Prompt and effective management of acute and chronic pain	1	*36 (46%)*	*17 (43%)*	*30 (48%)*
Dealing with a patient with psychiatric / psychological problems e.g. substance abuse, psychosis	1	44 (57%)	*13 (32%)*	*18 (29%)*
Working independently, where appropriate	2	44 (56%)	24 (59%)	41 (65%)
Being responsible for self-directed lifelong learning and professional development	2	**55 (71%)**	24 (59%)	**49 (78%)**
Teaching colleagues / students	2	45 (58%)	23 (58%)	**44 (70%)**
Being aware of your own limitations	2	**67 (86%)**	**34 (83%)**	**50 (79%)**
Managing your own health, including stress	2	46 (59%)	*20 (50%)*	*31 (49%)*
Prioritisation of tasks / time management	2	45 (58%)	*20 (49%)*	36 (57%)
Handover to colleagues	2	44 (56%)	25 (61%)	38 (60%)
Coping with responsibility	2	40 (51%)	24 (59%)	32 (51%)
Coping with uncertainty	2	*35 (46%)*	*19 (46%)*	*28 (45%)*
Discussing medication, including unwanted effects, with patients	3	**62 (80%)**	27 (66%)	**49 (79%)**
Discussing treatment options, including relative risks and benefits, with patients	3	**68 (87%)**	**33 (81%)**	**55 (89%)**
Agreeing on a satisfactory management plan with the involvement of your patient	3	**59 (76%)**	**29 (71%)**	**47 (76%)**

Taking opportunities to promote health and prevent disease	3	**62 (80%)**	**31 (76%)**	**44 (71%)**
Understanding the impact of the patient's condition on their psychological / emotional wellbeing	3	**72 (92%)**	**32 (78%)**	**51 (82%)**
Behaving with respect for patients	3	**75 (97%)**	**41 (100%)**	**56 (90%)**
Understanding the impact of the patient's social / cultural environment on their condition	3	**70 (90%)**	**34 (83%)**	**52 (84%)**
Taking an accurate drug history	4	**68 (87%)**	**32 (78%)**	**45 (71%)**
Examination skills	4	**70 (90%)**	**38 (93%)**	**59 (94%)**
History taking	4	**74 (95%)**	**39 (95%)**	**57 (91%)**
Diagnostic skills	4	53 (68%)	25 (61%)	43 (68%)
Understanding drug interactions	4	*18 (23%)*	*11 (27%)*	*22 (35%)*
Critical use of evidence (e.g. from audit, guidelines and research literature) in diagnosis and / or treatment	4	40 (52%)	*15 (37%)*	40 (65%)
Interpreting investigations	4	**57 (73%)**	22 (54%)	41 (65%)
Deciding which laboratory tests / investigations are required	4	46 (59%)	28 (68%)	39 (62%)
Requesting appropriate investigations (e.g. blood tests, radiology)	4	54 (69%)	27 (66%)	33 (52%)
Communicating with colleagues	5	**70 (90%)**	**36 (88%)**	**56 (89%)**
Communicating with patients' family / carers	5	**73 (94%)**	**36 (88%)**	**52 (83%)**
Communicating with patients	5	**75 (96%)**	**41 (100%)**	**60 (95%)**
Seeking help and advice from senior colleagues	5	**68 (87%)**	**35 (85%)**	**49 (78%)**
Working effectively in a multidisciplinary team	5	**72 (92%)**	**37 (90%)**	**49 (78%)**
Performing arterial puncture	6	**62 (80%)**	**29 (71%)**	**44 (71%)**
Performing urethral catheterisation	6	46 (59%)	26 (63%)	41 (67%)
Performing IV cannulation	6	**59 (76%)**	**35 (85%)**	**51 (82%)**
Prescribing drugs and treatments (including oxygen and fluids) appropriately and clearly	6	**59 (76%)**	28 (68%)	40 (65%)
Performing venepuncture	6	**69 (90%)**	**38 (93%)**	**55 (89%)**
Understanding medical ethical principles, including confidentiality and informed consent	7	**71 (91%)**	**35 (85%)**	**58 (92%)**
Practising infection control	7	**63 (81%)**	**39 (95%)**	**52 (83%)**
Understanding the legal framework of medical practice	7	**59 (76%)**	21 (51%)	**51 (81%)**
Making patient safety a priority in your clinical practice	7	**67 (86%)**	**36 (88%)**	**52 (83%)**

Verification of death / death certificate completion	8	52 (67%)	*16 (39%)*	33 (54%)
Completing discharge summaries	8	46 (59%)	*11 (27%)*	*24 (39%)*
Writing referral letters	8	*28 (36%)*	*10 (24%)*	*14 (23%)*
Keeping an accurate and pertinent medical record	8	**59 (76%)**	26 (63%)	37 (61%)

Subscale 1 - Treatment
Subscale 2 - Independent, responsible working
Subscale 3 - Dialoguing with patients
Subscale 4 - History, examination, diagnosis and investigation
Subscale 5 - Communication and team working
Subscale 6 - Procedural skills
Subscale 7 - Patient safety, ethics and legal issues
Subscale 8 - Paperwork

Table C: Mean (standard deviation) ratings of general preparedness, confidence in skills and knowledge and happiness at career choice

	**Medical School Type**			**Gender**		**Age Group**	
	**PBL (n = 78)**	**Reformed (n = 41)**	**Traditional (n = 63)**	**Male (n = 77)**	**Female (n = 105)**	**Age ≤24 (n = 88)**	**Age ≥ 25 (n = 93)**
Well prepared by experience at medical school *(1 = strongly disagree, 5 = strongly agree)*	4.07 (0.77)	3.78 (0.80)	3.82 (0.87)	3.88 (0.81)	3.94 (0.82)	4.09 (0.60)	3.75 (0.95)
	Not significant			*Not significant*		*$p < 0.05$*	
Confident that had necessary skills when starting F1 post *(1 = Not at all confident, 5 = Totally confident)*	2.64 (0.95)	2.42 (1.00)	2.59 (0.88)	2.73 (0.95)	2.45 (0.90)	2.73 (0.93)	2.41 (0.91)
	Not significant			*$p < 0.05$*		*$p < 0.05$*	
Confident that had necessary knowledge when starting F1 post *(1 = Not at all confident, 5 = Totally confident)*	2.41 (0.94)	2.42 (0.77)	2.83 (0.93)	2.78 (0.89)	2.37 (0.89)	2.71 (0.88)	2.39 (0.92)
	$p < 0.01$			*$p < 0.05$*		*$p < 0.05$*	
Happy to have chosen medicine as a career *(1 = strongly disagree, 5 = strongly agree)*	4.15 (1.01)	3.94 (0.75)	4.26 (0.73)	3.93 (0.99)	4.29 (0.75)	4.27 (0.78)	4.01 (0.94)
	Not significant			*$p < 0.05$*		*$p < 0.05$*	

Statistical tests used for comparing the various sub-groups: Medical School Type = Kruskal-Wallis; Gender = Mann-Whitney = Age Group: Mann-Whitney.
