# Supplementary material for: Medical graduates’ preparedness to practice: a comparison of undergraduate medical school training
Source: BMC Med Educ. 2017 Feb 6;17:33. doi: 10.1186/s12909-017-0859-6 (PMC5295184; doi:10.1186/s12909-017-0859-6)
Supplement: Additional file 2: — Preparation for working as an F1. This file is the full questionnaire used to collect data about preparedness for working as an F1. Data from the questions in Part 1 of the questionnaire are reported in this paper (please cite this paper if you use the questionnaire) and data from the questions in Part 2 of the questionnaire are reported in “Foundation doctors’ induction experiences by Susan Miles, Joanne Kellett, Sam J. Leinster. BMC Medical Education2015. 15:118. DOI: 10.1186/s12909-015-0395-1”. (PDF 690 kb) [file 12909_2017_859_MOESM2_ESM.pdf]

Preparation for working as an F1 - Aug/Sept 2011

Please note that if you complete and return this questionnaire you are giving us your consent to use the data you provide in the questionnaire for research purposes, which includes publication of your anonymised data in academic journals and other research publications. If you have any queries about this before completing the questionnaire, please feel free to contact NAME Research Associate. Email: EMAIL ADDRESS

This is a questionnaire for all first year Foundation (F1) doctors employed in posts with the East Anglian Foundation School. The purpose of the questionnaire is to find out how well or badly you think your medical student education and training has prepared you to work as an F1 doctor (Part 1); and your views on your Trust induction programme/s after qualifying as a doctor (Part 2). There is an optional personality test at the end (Part 3). If you would like to be entered into a prize draw to win one of five £50 Amazon.co.uk vouchers, please remember to provide the requested contact details at the end of the form. The prize draw will take place on Monday 12th December 2011 and winners will be notified by email after this date.

PART 1 - Your Medical Student Education and Training

1. Gender: Male ☐ Female ☐

2. Age: ☐

3. Name of University / Medical School where you studied: ☐

4. Year of qualification: ☐

5. Was this a graduate course? Yes ☐ No ☐

6. Did your medical student studies include time shadowing a foundation doctor? Yes ☐ No ☐

7. How well or badly do you think your *medical student education and training* has equipped you to be competent in the following areas?

a. Clinical skills

1) History taking	*Very badly* ☐	*Badly* ☐	*Slightly badly* ☐	*Slightly well* ☐	*Well* ☐	*Very well* ☐
2) Examination skills	*Very badly* ☐	*Badly* ☐	*Slightly badly* ☐	*Slightly well* ☐	*Well* ☐	*Very well* ☐
3) Diagnostic skills	*Very badly* ☐	*Badly* ☐	*Slightly badly* ☐	*Slightly well* ☐	*Well* ☐	*Very well* ☐
4) Deciding which laboratory tests / investigations are required	*Very badly* ☐	*Badly* ☐	*Slightly badly* ☐	*Slightly well* ☐	*Well* ☐	*Very well* ☐
5) Requesting appropriate investigations (e.g. blood tests, radiology)	*Very badly* ☐	*Badly* ☐	*Slightly badly* ☐	*Slightly well* ☐	*Well* ☐	*Very well* ☐
6) Interpreting investigations	*Very badly* ☐	*Badly* ☐	*Slightly badly* ☐	*Slightly well* ☐	*Well* ☐	*Very well* ☐
7) Taking an accurate drug history	*Very badly* ☐	*Badly* ☐	*Slightly badly* ☐	*Slightly well* ☐	*Well* ☐	*Very well* ☐
8) Understanding drug interactions	*Very badly* ☐	*Badly* ☐	*Slightly badly* ☐	*Slightly well* ☐	*Well* ☐	*Very well* ☐
9) Suggesting appropriate treatment for common symptoms e.g. nausea, pain etc.	*Very badly* ☐	*Badly* ☐	*Slightly badly* ☐	*Slightly well* ☐	*Well* ☐	*Very well* ☐
10) Critical use of evidence (e.g. from audit, guidelines and research literature) in diagnosis and / or treatment	*Very badly* ☐	*Badly* ☐	*Slightly badly* ☐	*Slightly well* ☐	*Well* ☐	*Very well* ☐

11) Dealing with a patient with airway problems	Very badly ☐	Badly ☐	Slightly badly ☐	Slightly well ☐	Well ☐	Very well ☐
12) Dealing with a patient with breathing problems e.g. acute asthma, pulmonary embolism	Very badly ☐	Badly ☐	Slightly badly ☐	Slightly well ☐	Well ☐	Very well ☐
13) Dealing with a patient with circulation problems e.g. hypotension, sepsis	Very badly ☐	Badly ☐	Slightly badly ☐	Slightly well ☐	Well ☐	Very well ☐
14) Dealing with a patient with neurological / visual problems e.g. seizures, coma	Very badly ☐	Badly ☐	Slightly badly ☐	Slightly well ☐	Well ☐	Very well ☐
15) Dealing with a patient with psychiatric / psychological problems e.g. substance abuse, psychosis	Very badly ☐	Badly ☐	Slightly badly ☐	Slightly well ☐	Well ☐	Very well ☐
16) Dealing with a patient with cognitive impairment e.g. dementia, delirium	Very badly ☐	Badly ☐	Slightly badly ☐	Slightly well ☐	Well ☐	Very well ☐
17) Dealing with an acutely unwell patient with complex needs e.g. medicine for the elderly	Very badly ☐	Badly ☐	Slightly badly ☐	Slightly well ☐	Well ☐	Very well ☐
18) Prompt and effective management of acute and chronic pain	Very badly ☐	Badly ☐	Slightly badly ☐	Slightly well ☐	Well ☐	Very well ☐
19) Responding effectively to emergencies	Very badly ☐	Badly ☐	Slightly badly ☐	Slightly well ☐	Well ☐	Very well ☐
20) Keeping an accurate and pertinent medical record	Very badly ☐	Badly ☐	Slightly badly ☐	Slightly well ☐	Well ☐	Very well ☐
21) Writing referral letters	Very badly ☐	Badly ☐	Slightly badly ☐	Slightly well ☐	Well ☐	Very well ☐
22) Completing discharge summaries	Very badly ☐	Badly ☐	Slightly badly ☐	Slightly well ☐	Well ☐	Very well ☐
23) Verification of death / death certificate completion	Very badly ☐	Badly ☐	Slightly badly ☐	Slightly well ☐	Well ☐	Very well ☐

b. Patient management

1) Agreeing on a satisfactory management plan with the involvement of your patient	Very badly ☐	Badly ☐	Slightly badly ☐	Slightly well ☐	Well ☐	Very well ☐
2) Behaving with respect for patients	Very badly ☐	Badly ☐	Slightly badly ☐	Slightly well ☐	Well ☐	Very well ☐
3) Discussing treatment options, including relative risks and benefits, with patients	Very badly ☐	Badly ☐	Slightly badly ☐	Slightly well ☐	Well ☐	Very well ☐
4) Discussing medication, including unwanted effects, with patients	Very badly ☐	Badly ☐	Slightly badly ☐	Slightly well ☐	Well ☐	Very well ☐
5) Understanding the impact of the patient's social / cultural environment on their condition	Very badly ☐	Badly ☐	Slightly badly ☐	Slightly well ☐	Well ☐	Very well ☐
6) Understanding the impact of the patient's condition on their psychological / emotional wellbeing	Very badly ☐	Badly ☐	Slightly badly ☐	Slightly well ☐	Well ☐	Very well ☐
7) Taking opportunities to promote health and prevent disease	Very badly ☐	Badly ☐	Slightly badly ☐	Slightly well ☐	Well ☐	Very well ☐

c. Practical procedures and skills

1) Performing venepuncture	Very badly ☐	Badly ☐	Slightly badly ☐	Slightly well ☐	Well ☐	Very well ☐
2) Performing IV cannulation	Very badly ☐	Badly ☐	Slightly badly ☐	Slightly well ☐	Well ☐	Very well ☐
3) Performing urethral catheterisation	Very badly ☐	Badly ☐	Slightly badly ☐	Slightly well ☐	Well ☐	Very well ☐
4) Performing arterial puncture	Very badly ☐	Badly ☐	Slightly badly ☐	Slightly well ☐	Well ☐	Very well ☐
5) Prescribing drugs and treatments (including oxygen and fluids) appropriately and clearly	Very badly ☐	Badly ☐	Slightly badly ☐	Slightly well ☐	Well ☐	Very well ☐

d. Communication and team-working

1) Communicating with patients	Very badly ☐	Badly ☐	Slightly badly ☐	Slightly well ☐	Well ☐	Very well ☐
2) Communicating with patients' family / carers	Very badly ☐	Badly ☐	Slightly badly ☐	Slightly well ☐	Well ☐	Very well ☐
3) Communicating with colleagues	Very badly ☐	Badly ☐	Slightly badly ☐	Slightly well ☐	Well ☐	Very well ☐
4) Working effectively in a multidisciplinary team	Very badly ☐	Badly ☐	Slightly badly ☐	Slightly well ☐	Well ☐	Very well ☐
5) Seeking help and advice from senior colleagues	Very badly ☐	Badly ☐	Slightly badly ☐	Slightly well ☐	Well ☐	Very well ☐
6) Handover to colleagues	Very badly ☐	Badly ☐	Slightly badly ☐	Slightly well ☐	Well ☐	Very well ☐
7) Teaching colleagues / students	Very badly ☐	Badly ☐	Slightly badly ☐	Slightly well ☐	Well ☐	Very well ☐

e. Clinical guidelines and protocols

1) Making patient safety a priority in your clinical practice	Very badly ☐	Badly ☐	Slightly badly ☐	Slightly well ☐	Well ☐	Very well ☐
2) Understanding the legal framework of medical practice	Very badly ☐	Badly ☐	Slightly badly ☐	Slightly well ☐	Well ☐	Very well ☐
3) Understanding medical ethical principles, including confidentiality and informed consent	Very badly ☐	Badly ☐	Slightly badly ☐	Slightly well ☐	Well ☐	Very well ☐
4) Practising infection control	Very badly ☐	Badly ☐	Slightly badly ☐	Slightly well ☐	Well ☐	Very well ☐

f. Personal development and wellbeing

1) Being aware of your own limitations	Very badly ☐	Badly ☐	Slightly badly ☐	Slightly well ☐	Well ☐	Very well ☐
2) Prioritisation of tasks / time management	Very badly ☐	Badly ☐	Slightly badly ☐	Slightly well ☐	Well ☐	Very well ☐
3) Being responsible for self-directed lifelong learning and professional development	Very badly ☐	Badly ☐	Slightly badly ☐	Slightly well ☐	Well ☐	Very well ☐
4) Managing your own health, including stress	Very badly ☐	Badly ☐	Slightly badly ☐	Slightly well ☐	Well ☐	Very well ☐
5) Coping with responsibility	Very badly ☐	Badly ☐	Slightly badly ☐	Slightly well ☐	Well ☐	Very well ☐
6) Coping with uncertainty	Very badly ☐	Badly ☐	Slightly badly ☐	Slightly well ☐	Well ☐	Very well ☐
7) Working independently, where appropriate	Very badly ☐	Badly ☐	Slightly badly ☐	Slightly well ☐	Well ☐	Very well ☐

8. Please use this space if you would like to provide any comments about how prepared you felt in any of the areas listed above:

Next, there are a few questions about your experiences generally…

9. Overall, my experience at medical school prepared me well for the tasks I have undertaken so far as an F1 doctor:

Strongly disagree ☐ Disagree ☐ Neutral ☐ Agree ☐ Strongly agree ☐

10. Please try to think back to when you started your F1 post, how confident did you feel that you had the necessary skills to do your F1 job?

Not at all confident ☐ Slightly confident ☐ Moderately confident ☐ Very confident ☐ Totally confident ☐

11. Please try to think back to when you started your F1 post, how confident did you feel that you had the necessary knowledge to do your F1 job?

Not at all confident ☐ Slightly confident ☐ Moderately confident ☐ Very confident ☐ Totally confident ☐

12. Please list any skills or knowledge that you have needed during your F1 post so far which were not adequately covered by your medical training:

13. Please provide details of how your medical training could have been done differently so that you were more prepared for your F1 post:

14. We are interested in knowing about any difficulties you have encountered in your daily work as an F1 doctor due to being unprepared. If you have experienced any particular problems, please describe below:

PART 2 - Your Hospital / Departmental Induction

1) Please list the placements you have undertaken during your F1 post so far:

a) Department: ____________________

b) Trust: ____________________

These next questions are about your departmental induction; i.e. the introduction you received to your department, its procedures and personnel. Not your hospital induction or Preparation for Professional Practice (PfPP)…

2. How long was your departmental induction (i.e. the introduction to your department, its procedures and personnel)

Less than 1 hour ☐ 1 – 2 hours ☐ 2 – 4 hours ☐ 4 hours – 1 day ☐

More than 1 day ☐ N/A induction not provided ☐ N/A did not attend induction ☐

3. How useful were the following elements of your departmental induction/introduction *(if you didn't have a departmental induction please go to question 4 below)*

	Not at all useful	*Moderately useful*	*Extremely useful*	***Not covered***
Introduction to key members of the department				
Regular duties of the job				
What is expected of you as an F1 in the department				
Departmental policies/guidelines & how to access them				
Consultant preferences for patient management				
Ordering tests and related paperwork				
Discharge policies and related paperwork				
Referral policies				
Who to contact in different departments				
Handover policies				
Senior support and how to contact in day				
Senior support and how to contact at night				
Annual leave policies				
Study leave policies				
Rota				
Teaching sessions				
Tour of department - where is everything?				
Cardiac arrest and where is the trolley?				

4. Is your departmental handbook useful?

Yes ☐ No ☐ N/A no handbook ☐

Thinking now about all of the induction experiences you have had since starting your F1 post…

5. How do you rate each of these from the perspective of how helpful they were in preparing you for your role as an F1 doctor? *(Please use the 7-point scale, where 1 means that the induction was No help at all, and 7 means that the induction was Extremely helpful i.e. a higher number indicates the induction was more helpful)*

	No help at all						*Extremely helpful*	*N/A or Don't remember*
**a. Preparation for Professional Practice (PfPP)**	**1**	**2**	**3**	**4**	**5**	**6**	**7**	
**b. Hospital induction**	**1**	**2**	**3**	**4**	**5**	**6**	**7**	
**c. Departmental induction / introduction**	**1**	**2**	**3**	**4**	**5**	**6**	**7**	

6. Please tell us the most useful aspect/s of your Trust / PfPP induction programmes:

7. Please tell us the most useful aspect/s of your departmental induction (i.e. the introduction to your department, its procedures and personnel) if provided:

8. In your opinion, what gaps were there in the induction you received?

9. If you have any suggestions for how these gaps could be filled then please provide them here:

10) Please rate your agreement with the following statement "Generally, I am happy that I chose medicine as a career":

Strongly disagree ☐ Disagree ☐ Neutral ☐ Agree ☐ Strongly agree ☐

11. Any other comments about how you could have been better prepared for your F1 role?

PART 3 - About You

Finally, we would appreciate it if you could take the time to complete the following 11 questions. If you do not wish to answer these questions, you can stop now. But remember to provide your contact details (see below) first if you wish to be entered into the prize draw

I see myself as someone who…….	*Disagree strongly*	*Disagree a little*	*Neutral*	*Agree a little*	*Agree strongly*
… is reserved					
… is generally trusting					
… tends to be lazy					
… is relaxed, handles stress well					
… has few artistic interests					
… is outgoing, sociable					
… tends to find fault with others					
… does a thorough job					
… gets nervous easily					
… has an active imagination					
… is considerate and kind to almost everyone					

That's it! Thank you very much for your time. Please send the completed form to the research team in the enclosed FREEPOST envelope.

If you wish to enter the prize draw to win a £50 Amazon.co.uk voucher please provide the following contact details:

Name:	
Trust:	
Email address:<br>(if you win, your voucher will be emailed to this address, so please check it's accurate / legible)	

The personal data you provide here will not be used for any purpose other than the prize draw, and will be kept separate from the other data you have provided on the questionnaire.
